# Can the Novel Photon-Counting CT Scan Accurately Predict Aortic Wall Thickness? Preliminary Results

**DOI:** 10.3390/bioengineering12030306

**Published:** 2025-03-18

**Authors:** Alessandra Sala, Carlo de Vincentiis, Francesco Grimaldi, Barbara Rubino, Manuela Cirami, Noemi Perillo, Renato Vitale, Rosanna Cardani, Sara Boveri, Michele Conti, Pietro Spagnolo

**Affiliations:** 1 Department of Cardiac Surgery, IRCCS Policlinico San Donato, San Donato Milanese, 20097 Milan, Italy; alessandra.sala@grupposandonato.it (A.S.); francesco.grimaldi@grupposandonato.it (F.G.); 2 Department of Pathology, IRCCS Ospedale Galeazzi-Sant’Ambrogio, 20157 Milan, Italy; barbara.rubino@grupposandonato.it (B.R.); manuela.cirami@grupposandonato.it (M.C.); 3 Department of Radiology, IRCCS Policlinico San Donato, San Donato Milanese, 20097 Milan, Italy; noemi.perillo@grupposandonato.it (N.P.); renato.vitale@grupposandonato.it (R.V.); pietro.spagnolo@grupposandonato.it (P.S.); 4 BioCor-Biobank, IRCCS Policlinico San Donato, San Donato Milanese, 20097 Milan, Italy; rosanna.cardani@grupposandonato.it; 5 Laboratory of Biostatistics and Data Management, Scientific Directorate, IRCCS Policlinico San Donato, San Donato Milanese, 20097 Milan, Italy; sara.boveri@grupposandonato.it; 6 Department of Civil Engineering and Architecture, Structural Mechanics Division, Università Degli Studi di Pavia, 27100 Pavia, Italy; michele.conti@unipv.it; 7 3D and Computer Simulation Laboratory, IRCCS Policlinico San Donato, San Donato Milanese, 20097 Milan, Italy

**Keywords:** aortic wall, aortic aneurysm, photon-counting CT scan, aortic wall thickness, thoracic aorta, cardiac surgery

## Abstract

Background: Surgical indication of ascending thoracic aortic aneurysms (ATAA) is generally performed in prevention. Guidelines use aortic diameter as a predictor of rupture and dissection; however, this single parameter alone has a limited value in predicting the real-world risk of acute aortic syndromes. The novel photon-counting CT scan(pc-CT) is capable of better-analyzing tissue composition and aortic characterization. The aim of the study is to assess whether the correlation between aortic wall thickness measured with a pc-CT scan and histology exists. Methods: 14 Patients, with a mean age of 47 years, undergoing cardiac surgery for ATAA, who had preoperatively undergone a pc-CT scan, were retrospectively analyzed. Histology analyses of the resected aortic wall aneurysm were reviewed, and minimum/maximum measurements of intima+media of the aortic wall were performed. Radiology images were also examined, and aortic wall thickness measures were taken. Bland-Altman plots and Passing-Bablock regression analyses were conducted to evaluate the correlation between the values. Results: pc-CT scan mean measurements were 1.05 and 1.69 mm, minimum/maximum, respectively. Mean minimum/maximum histology measurements were 1.66 and 2.82 mm, respectively. Bland Altman plots and Passing-Bablock regression analyses showed the absence of systematic bias and confirmed that measurement values were sufficiently similar (minimum −0.61 [CI 95% 0.16–1.38]; maximum −1.1 [0.73–2.99]). Conclusions: Despite results being merely preliminary, our study shows encouraging sufficiently similar results between aortic wall thickness measurements made with pc-CT scan and histology analyses.

## 1. Introduction

The aorta is a large vessel that delivers oxygenated blood to the entire body. The wall comprises three layers: the intima, the media, and the peripheral adventitia, which all contribute differently to the mechanical properties of the aorta [1]. The aortic wall is subjected to mechanical stresses in radial, circumferential, and longitudinal directions [2]. Circumferential stress predominantly affects the media, leading to medial degeneration and the development of the most common thoracic aortic pathology: aortic aneurysms [3,4].

Thoracic aortic aneurysms (TAA) occur in 5–10 per 100,000 person-years, and approximately 60% involve the ascending thoracic aorta [5]. Most are incidentally diagnosed on thoracic imaging, as only 5% of patients are symptomatic on presentation.

Surgical indication is performed in prevention. Guidelines use aortic diameter and its growth rate as predictors of rupture and dissection, which is the result of the aortic tissue ceasing to withstand mechanical stress and resulting in a potentially lethal emergency, with pre-hospital mortality of 40% [6]. Studies have identified a cut-off diameter of approximately ≥ 5.0–5.2cm (American [7]—European guidelines [8]) as the correct timing for elective aortic repair to avoid rupture or dissection.

However, nearly 40% of patients presenting with acute aortic syndrome have aortic diameters < 5.0 cm [9]. Therefore, diameter alone has a limited value in predicting the real-world risk of acute aortic rupture or dissection because dilation is only one of many manifestations of structural weakness of the aortic wall.

Hence, diameter alone is a poor marker for risk stratification, and surgery based on aortic size alone will prevent only a minority of aortic dissections [10,11].

This unmet clinical need has led to the interest and necessity in identifying other parameters capable of further characterizing the aorta and providing added tools to the evaluation of patient-specific aortic aneurysms.

Computed tomography (CT) has been established as the gold-standard diagnostic tool for thoracic aortic aneurysms due to its high-resolution, 3-dimensional (3D) image data sets and rapid acquisition. The most recent CT technology, dual-source photon-counting, has the capability of increasing the spatial resolution of images without electronic noise and, therefore, provides improved image contrast [12,13,14]. Due to the increased imaging precision, this novel technology may analyze various aspects of the aorta in-depth, such as aortic wall thickness, and provide an additional parameter capable of further characterizing the aorta [15]. This preoperative factor, detectable with a routing photon-counting CT scan, may provide another tool to our toolbox, playing an essential role in patient stratification.

The study aims to analyze patients undergoing cardiac surgery, compare preoperative CT scan images and in-vitro data regarding aortic wall thickness, and determine whether a preliminary correlation exists between the obtained values.

## 2. Materials and Methods

### 2.1. Study Population

A single-centre retrospective study, including patients undergoing cardiac surgery for either aortic root/ascending aortic aneurysm or aortic valve disease with normal ascending aorta, who had preoperatively undergone photon-counting CT scan from November 2023 to March 2024, in one cardiac surgery center in Milan, IRCCS Policlinico San Donato, was conducted. All consecutive patients were individually reviewed, and patients who had previously undergone cardiac surgery were excluded. Among the total population, 14 patients were identified.

**Figure 1:**
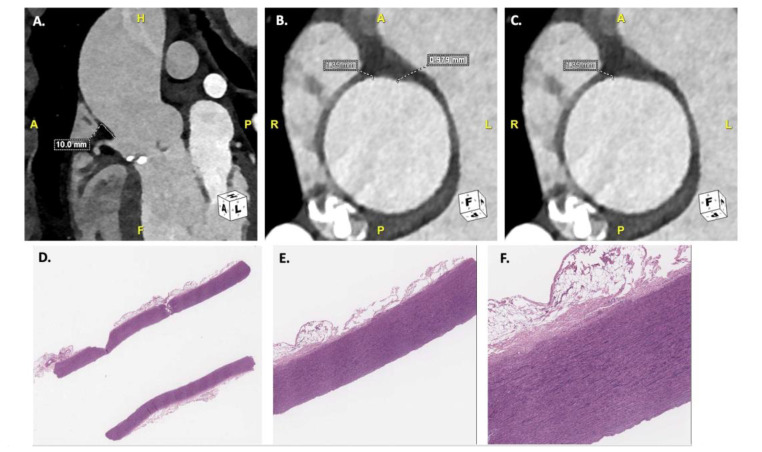
CT scan images (**A**–**C**) and histology images (**D**–**F**) used to perform aortic wall thickness measurements. (**A**) defining height from right coronary artery ostium for correct measurement; (**B**,**C**) aortic wall thickness measurements, considering minimum and maximum values; (**D**) 2.5× magnification of histology analysis, with hematoxylin-eosin coloration; (**E**) 10× magnification; (**F**) 20× magnification.

Charts were reviewed to identify preoperative characteristics, laboratory values, and echocardiographic and CT parameters. All patients had undergone preoperative transthoracic echocardiography (TTE) upon hospitalization. Aortic valve anatomy was reported as bicuspid or tricuspid according to preoperative TTE imaging, CT scan imaging, and intra-operative findings. All patients had also performed a preoperative photon-counting CT scan as a routine workup, identifying aortic root/ascending aortic dimensions, aneurysm anatomy, and coronary artery anatomy. Patients underwent ECG-gated UHR-CTA of the aortic root and cardiac structures (collimation: 120 × 0.2 mm), with a temporal resolution of 66ms, during a breath hold, directly followed by a non-ECG-synchronized CTA of the thorax, abdomen, and pelvis (collimation: 144 × 0.4 mm). A retrospective review of the preoperative photon-counting CT scan images also provided aortic wall thickness measurements in the diastolic phase, taking into consideration intima and media portions of the aortic wall, ranging from a minimum to a maximum thickness measured, based on the landmark provided by the resected material during surgery (Figure 1). Measurements were performed by two radiologists, and the average between measurements, both minimum and maximum, was considered for the study. The diagnostic quality and interpretability of aortic root CT scan images were graded by two experienced radiologists using the following semiquantitative 4-point scale: 1—bad (insufficient opacification or severe motion artifacts); 2—poor (inhomogeneous enhancement with markedly blurred vessel edges, pronounced motion artifacts); 3—adequate (homogeneous enhancement with moderately blurred vessel edges, only minor motion artifacts); 4—good (homogeneous enhancement with good visibility of the anatomic details, no motion artifacts). Images classified as “good” and “adequate” were considered evaluable/diagnostic. Disagreements in data analysis between the two observers were resolved by consensus reading.

Intraoperative and postoperative characteristics, together with postoperative echocardiographic data, were also analyzed, and all data were inserted within an anonymous and dedicated database.

Histological analyses of the patients treated were also retrospectively reviewed. Aortic biopsies derive from biological material removed during cardiac surgery due to pathological indications. The resected biological material was marked; height from the ostium of the right coronary artery was used as a landmark to guide precise and equal measurements taken from the CT scans and in-vitro testing. The sample was placed in 10% neutral buffered formalin and sent to the Department of Pathology for routine histological analysis as per standard clinical practice. For each patient, a full-thickness sample of the aortic wall was taken, processed overnight, and embedded in paraffin. Hematoxylin-eosin-stained slides were prepared for each inclusion and morphologically assessed under light microscopy (Figure 1). Subsequently, the slide was digitally scanned, and measurements were then taken in millimeters of the aortic wall thickness at the point of minimum and maximum thickness.

### 2.2. Outcomes

The primary endpoint of the study was a preliminary evaluation of the preoperative photon-counting CT scan measurement of the aortic wall thickness and in-vitro histological analysis of the aortic wall thickness to try and define a preliminary correlation between the values obtained.

### 2.3. Statistical Analyses

Analyses were exploratory in nature. Clinical and preoperative photon-counting CT scan measurements were described with numbers and frequencies for categorical data and with mean ± standard deviation or median and interquartile range for continuous data, according to the distribution of the variable. The normality assumption was tested by visual inspection of the a-plot and with the Shapiro-Wilk test.

The precision of the estimate was assessed using Bland-Altman, with the estimate’s precision with 95% limits of agreement. The formula for calculating a 95% confidence interval for the limits of agreement was: *n* = 3(2 × 1.96 × s/lw)^2^ [16]. A sample size of 14 patients was obtained to estimate a confidence interval with a half-length equal to the standard deviation. Statistical significance will be set at a probability value of less than or equal to 0.05. Power analysis was not performed due to the retrospective nature of the study and the lack of evidence that retrospective power can be accurately and effectively calculated [17].

Statistical analyses were performed using SAS version 9.4 (SAS Institute, Cary, NC, USA).

### 2.4. Data Availability

All data will be available upon request.

## 3. Results

### 3.1. Patients’ Characteristics

The baseline characteristics of all patients are listed in Table 1.

Among the population, 12 patients (85.7%) underwent surgery for ascending thoracic aortic aneurysm, and only one patient underwent isolated aortic valve replacement (AVR), while the other underwent aortic valve repair (AVr). The mean age of the study group was 47.6 ± 11 years, and the most common cardiovascular risk factors were hypertension (57%) and family history of aortic diseases (21%). Only one patient had a preoperative diagnosis of Marfan syndrome.

More than half of patients (57%) had at least moderate aortic regurgitation (AR), while 21.4% of patients had severe aortic stenosis (AS). 8/14 (57.1%) patients had bicuspid aortic valves upon preoperative echocardiography and upon intra-operative inspection. Left ventricular ejection fraction (EF) was preserved (59.1 ± 7.3%), with good right ventricular function and no associated concomitant valvular or coronary artery diseases. Upon preoperative CT scan, mean aortic root dimensions were 40.7 ± 10.1 mm, and mean ascending aortic diameters were 44.9 ± 6.9 mm, with normal aortic arch and descending thoracic aorta (Table 2).

Intraoperatively, 4 patients (28.6%) underwent aortic root replacement surgery with a mechanical-valved conduit (Bentall procedure); a valve-sparing root replacement surgical technique (T. David technique) was preferred in two patients, while aortic valve and ascending aortic replacement was performed in 5 patients (35.7%). Furthermore, one patient underwent isolated AVR with a biological prosthesis, one patient underwent isolated AVr with right coronary cusp plication and commissural triangles resuspension, and one patient underwent isolated ascending aortic replacement with vascular graft. Mean cardiopulmonary bypass times were 85.1 ± 25.8 min, and aortic cross-clamp times 71.5 ± 22.8 min (Table 3).

Postoperative outcomes were regular in all cases, with good surgical results. All implanted prostheses functioned adequately postoperatively, and all aortic valve repairs had good outcomes, with minimal aortic regurgitation in one case. All patients were discharged home with good biventricular function and no need for pacemaker implantation.

### 3.2. Aortic Wall Thickness Measurements

All patients underwent retrospective review and analyses of preoperative photon-counting CT scan images. Image quality was independently scored to be diagnostic by both readers in all cases: it was rated as being “good” in 12 scans (85.7%) and “adequate” in the remaining two scans (14.3%). The mean image quality score was 3.86 ± 0.36 [range 3–4]. Aortic wall thickness was measured at the indicated height from the right coronary artery ostium, and measurements of total aortic wall thickness in diastole, comprising intima + media, were performed. Minimum and maximum measurements were reported. Regarding minimum thickness, a mean of 1.05 ± 0.40 mm and a median of 1.06 mm [0.90–1.11] were measured; the mean maximum measurements were 1.69 ± 0.59 mm and a median of 1.79 mm [1.30–1.89].

The in-vitro samples, stored in formalin and processed overnight, were also thoroughly analyzed to define total aortic wall thickness, comprising intima and medial layers. The mean minimum histological measurement was 1.66 ± 0.50 mm, with a median of 1.75 mm [1.20–1.90], while the mean maximum and median measurements were 2.82 ± 0.95 mm and 2.85 mm [2.20–3.60], respectively (Table 4).

**Figure 2:**
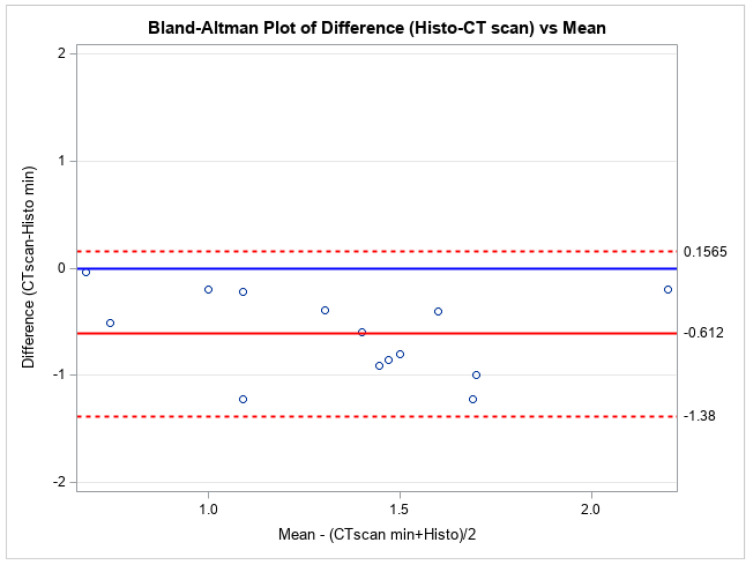
Bland-Altman Plot of the minimum measurements.

**Figure 3:**
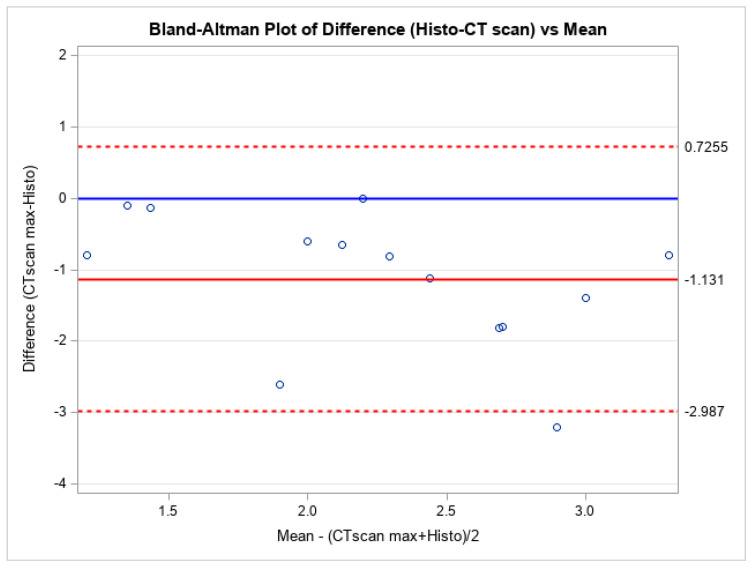
Bland-Altman Plot of the maximum measurements.

A Bland-Altman plot of differences for both minimal and maximal measurements compared to their average was performed (Figure 2 and Figure 3). The plots show no systematic bias or error. Further measurements were analyzed through Passing and Bablock regression analysis (Table 5), which confirmed that the photon-counting CT scan yields similar measurements to the histological thickness values. To quantify the uncertainty of the estimated limits of agreement, we included in the study the width of the confidence intervals (upper agreement limit CI: (0.537, −0.232); lower agreement limit CI: (−0.991, −1.761)).

## 4. Discussion

This retrospective, single-center study aimed at assessing the ability of the novel photon-counting CT scan to accurately measure the thickness of the aortic wall in patients undergoing cardiac surgery for aortic root/ascending aortic aneurysm or aortic valve disease with normal ascending aorta.

Recent European guidelines have modified the diameter cut-off value for surgical indication, lowering the threshold to 5.0–5.2 cm [8]. Guidelines are constantly changing, and precise indications are lacking. Surgery for this pathology is performed in prevention to avoid acute aortic events, such as aortic wall rupture or dissection. Older studies had identified 6 cm as the hinge point above which the risk of acute aortic syndromes would increase exponentially, with a yearly risk of 15% [18]. More recent analyses have identified a smaller diameter, at 5.0–5.5 cm, as the pivot above which the risk increases [19].

Furthermore, within the aortic guidelines, other risk factors have been listed that play a role in favoring surgical indication, such as the length of the ascending aorta, a significant growth rate per year, arterial hypertension, a small height, and young age [8]. However, nearly 40% of patients presenting with acute aortic syndromes have aortic diameters < 5.0 cm [9]. Therefore, diameter alone has a limited value in predicting the real-world risk of acute aortic rupture or dissection because dilation is only one of many manifestations of structural weakness of the aortic wall. Despite the added risk factors, diameter alone is a poor marker for risk stratification in ascending aortic aneurysms, and surgery based on aortic size alone will prevent only a minority of aortic dissections [11]. There is a growing interest in and necessity for identifying another parameter capable of further characterizing the aorta and providing added tools to evaluate aortic aneurysms and surgical timing.

Tissue mechanical stress in the vessel wall is a function of radius and wall thickness. Therefore, the role of wall thickness in ascending aortic aneurysm formation in clinical practice is worthy of proper attention/importance. ATAA is characterized by disproportionate degeneration of the media compared to the healthy aorta, with the remodeling of the extracellular matrix leading to fewer vascular smooth cells and the degradation of elastin fibers [3]. However, the effects of wall thickness changes during aneurysms are ill-defined. Also, given the notion that wall thickness is a determinant of mechanical homeostasis, there is a clear need for consistent and clinically applicable methods and studies to quantify wall thickness in ascending aortic aneurysms.

The novel dual-source photon-counting CT scan has the ability to increase spatial resolution, reduce electric noise, and better characterize the aortic wall. However, its ability to define aortic wall thickness has never been studied, to the best of our knowledge, to date. Preoperative CT scan, compared to other imaging modalities such as magnetic resonance imaging (MRI), is our standard of practice in order to assess preoperatively the status of the coronary arteries as well as obtain adequate patient planning, therefore resulting in a cost-effective imaging exam.

Our study, even though simply preliminary, on a minimal number of patients, showed comparable measurements of aortic wall thickness between preoperative photon-counting CT scans and postoperative in-vitro histologic measurements. As shown, following the Passing-Bablock regression analysis, measurements were considered similar. However, looking more closely into the measurements obtained, there was a minimal, non-statistically significant underestimation of the aortic wall thickness with CT scan images. Despite formalin fixation being known to cause shrinkage of the studied material [20], a couple of aspects should be considered that may justify our findings.

First and foremost, preoperative CT scan analyses evaluate the in-vivo aortic wall thickness in an anatomical area rich in elastic components. The intraluminal pressure from the blood causes the aortic wall to expand, which can lead to a thinner wall when analyzed ex vivo.

Secondly, the potential alteration of the fixed tissue in formalin may have a minimal impact on the measurements taken. In fact, the effect of formalin on various tissues differs based on their composition and the duration of formalin fixation. Some studies have shown that prolonged formalin fixation (greater than 168 h) results in minimal or insignificant changes in tissue dimensions compared to non-preserved samples [21].

Also, the Bland-Altman statistical analyses performed showed no systematical bias or error that may be justified by the conservation of the material.

Nowadays, computed tomography is considered the gold-standard diagnostic tool, and patients with ascending aorta dilation undergo regular follow-ups with CT scans. This ability of the novel photon-counting CT scans to better characterize the aorta may provide a preoperative non-invasive assessment of aortic wall thickness as an additional parameter within the evaluation of patients with thoracic aortic aneurysm pathology. This further information will require additional studies for a more complete and accurate correlation with aneurysm formation and risk prediction. Further studies will require more in-depth analysis of aortic wall thickness, associated with diameter, aortic length, strength, and tensile characteristics of the aortic tissue, as well as hypertension tendency, in order to provide a wide panel of parameters capable of increasing the knowledge of the pathology and provide all the tools to define surgical indication.

## 5. Conclusions

Even though it is simply preliminary in nature, our study shows that the novel photon-counting CT scan is capable of determining preoperatively the aortic wall thickness in patients with either aortic root/ascending aortic aneurysms or normal ascending aorta. The samples examined were limited in number. Therefore, significant studies will be required in order to confirm our preliminary results and confidently say that the measurements obtained are accurate and precise. Our institution is planning to start a prospective single-center study in order to reach an adequate sample size that will confirm these findings. Furthermore, two other limitations of the present study, related to its retrospective nature and preliminary design, are the lack of inter- and intra-observer variability calculation for the measurements taken and the lack of a power analysis. Both aspects will be thoroughly addressed in the upcoming prospective study in order to further validate such results. However, sufficiently similar measurements were reported between photon-counting CT scans and histology, laying the foundations for further research.

## Figures and Tables

**Table 1:** Preoperative Patients’ Characteristics.

Characteristics	N (%) or Mean ± STD − Median [IQR]
**Patients’ characteristics**	
Age (years)	47.57 ± 10.98 − 46 [39–58]
Gender (M)	11 (78.57)
BMI (kg/m^2^)	24.16 ± 2.75 − 24.30 [22.80–25.90]
BSA (m^2^)	1.85 ± 0.20 − 1.86 [1.75–2.01]
EuroSCORE (%)	1.12 ± 0.35 − 1 [0.96–1.30]
**Risk Factors**	
Hypertension	8 (57.14)
CKD	2 (14.29)
Dyslipidemia	3 (21.43)
DM	1 (7.14)
OSAS + CPAP	1 (7.14)
Family history aortic disease	3 (21.43)
Current smoker	4 (28.57)
Former smoker	3 (21.43)
**NHYA**	
I	11 (78.57)
II	3 (21.43)
**Pathology**	
Ascending aortic aneurysm	8 (57.14)
Root aneurysm	5 (35.71)
No aneurysm	2 (14.29)
**Medical therapy**	
ACE-inhibitors	2 (14.29)
ARBs	4 (28.57)
Ca-antagonist	2 (14.29)
Cardioaspirin	2 (14.29)
β-blockers	5 (35.71)
Diuretics	1 (7.14)

ARBs: angiotensin receptor blockers; BMI: body mass index; BSA: body surface area; Ca: calcium; CKD: chronic kidney disease; CPAP: continuous positive ventilation pressure; DM: diabetes mellitus; IQR: interquartile range; NYHA: New York Heart Association; OSAS: obstructive sleep apnea syndrome; STD: standard deviation.

**Table 2:** Preoperative Imaging Exams.

Characteristics	N (%) or Mean ± STD − Median [IQR]
**Echocardiography**	
LVEDD (mm)	50.36 ± 7.57 − 48.50 [44–54]
LVEDV (mL)	144.79 ± 52.95 − 138 [96–156]
LVEF (%)	59.07 ± 7.28 − 58.50 [53–65]
RVEDD (mm)	34.93 ± 2.53 − 35 [34–37]
TAPSE (mm)	23.38 ± 2.81 − 23.50 [21–25]
*AR*	
0–1+	6 (42.86)
2+	1 (7.14)
3–4+	7 (50)
*AS*	
0–1+	10 (71.43)
2+	1 (7.14)
3–4+	3 (21.43)
AV Gmed (mmHg)	16.79 ± 17.11 − 7 [5–28]
AV Gmax (mmHg)	25.86 ± 24.96 − 13 [9–44]
Aortic root (mm	41.86 ± 9.41 − 39.50 [37–48]
Ascending aorta (mm)	45.07 ± 7.92 − 44.50 [40–49]
**CT scan**	
Aortic root (mm)	40.71 ± 10.12 − 39.50 [35–44]
STJ (mm)	39.36 ± 7.42 − 36.50 [35–45]
Ascending aorta (mm)	44.89 ± 6.91 − 45.50 [42–51]
Aortic arch (mm)	27.93 ± 3.89 − 28 [25–30]
Descending thoracic aorta (mm)	25.39 ± 3.69 − 25.50 [22–26]

AR: aortic regurgitation; AS: aortic stenosis; AV: aortic valve; CT: computed tomography; Gmax: maximum gradient; Gmed: median gradient; IQR: interquartile range; LVEDD: left ventricular end diastolic diameter; LVEDV: left ventricular end diastolic volume; LVEF: left ventricular ejection fraction; RVEDD: right ventricular end diastolic diameter; STD: standard deviation; STJ sino-tubular junction; TAPSE: tricuspid annular plane systolic excursion.

**Table 3:** Surgical Data.

Characteristics	N (%) or Mean ± STD − Median [IQR]
**Intraop AV inspection**	
Bicuspid	8 (57.14)
Tricuspid	6 (42.86)
**Surgical procedure**	
AVR	10 (71.43)
AVr	1 (7.14)
Bioprosthesis	1 (7.14)
Bentall	4 (28.57)
T. David	2 (14.29)
Wheat	5 (35.71)
**Surgical times**	
CPB (min)	85.07 ± 25.78 − 84 [64–103]
XCT (min)	71.50 ± 22.80 − 71.50 [53–85]

AV: aortic valve; AVr: aortic valve repair; AVR: aortic valve replacement; CPB: cardiopulmonary bypass; IQR: interquartile range; STD: standard deviation; XCT: cross-clamp time.

**Table 4:** Aortic wall thickness measurements.

Measurements	Histology	Photon-Counting CT Scan
Minimum (mm)	1.66 ± 0.50 − 1.75 [1.20–1.90]	1.05 ± 0.40 − 1.06 [0.90–1.11]
Maximum (mm)	2.82 ± 0.95 − 2.85 [2.20–3.60]	1.69 ± 0.59 − 1.79 [1.30–1.89]

Data are reported as mean ± standard deviation or median [interquartile range].

**Table 5:** Passing-Bablock Regression analysis.

Measurements	α	β	Interpretation
Minimum	0.06 [−0.92−0.67]	0.58 [0.22–1.24]	Measurements are similar
Maximum	0.63 [−0.84–1.26]	0.45 [0.17–1.01]	Measurements are similar

## Data Availability

All data will be available upon request from the authors.
